# Prevalence of smoking and other smoking-related behaviors reported by the Global Youth Tobacco Survey (GYTS) in Thailand

**DOI:** 10.1186/1471-2458-8-S1-S3

**Published:** 2008-12-15

**Authors:** Nithat Sirichotiratana, Chairat Techatraisakdi, Khalillur Rahman, Charles W Warren, Nathan R Jones, Samira Asma, Juliette Lee

**Affiliations:** 1 Health Administration Department, Faculty of Public Health, Mahidol University, 420/1 Rajvithi Road, Rajthevi District, Bangkok 10400, Thailand; 2 Director of Diseases Control Region 1, Diseases Control Department, Ministry of Public Health, Tivanont Road, Nontaburi 11000, Thailand; 3 Regional Advisor/Tobacco Free Initiative, WHO-South-East Asia Regional Office, New Delhi 110002, India; 4 Office on Smoking and Health, Centers for Disease Control and Prevention, 4770 Buford Highway NE, Atlanta, Georgia 30341, USA

## Abstract

**Introduction:**

Thailand ratified the World Health Organization (WHO) Framework Convention on Tobacco Control (FCTC) on November 8, 2004. The WHO FCTC requires all parties to inform all persons of the health consequences of tobacco consumption and exposure to tobacco smoke. Each party has agreed to develop, implement and evaluate effective tobacco control programs to measure progress in reaching the goals of the WHO FCTC.

**Methods:**

The Global Youth Tobacco Survey (GYTS) was developed to provide data on youth tobacco use to countries for their development of youth-based tobacco control programs. Data in this report can be used as baseline measures for future evaluation of the tobacco control programs implemented by the Ministry of Public Health.

**Results:**

Overall, about 1 in 10 Thai students are current smokers, this number including 4 times more boys than girls (17% versus 3.9%). Almost 2 in 10 Thai students start smoking before the age of 10, and almost 7 in 10 students are reported to have been exposed to smoke from others in public places. About 4 in 10 students are reported to have an object with a cigarette brand logo on it.

**Conclusion:**

The key for Thailand is to implement and enforce the provisions on indirect tobacco advertising, smoking in public places, selling tobacco to youths under 18 years of age, and to use the data from the GYTS to monitor progress toward achieving the goals of the WHO FCTC. When these goals are met, tobacco consumption and exposure in Thailand will have declined substantially.

## Introduction

The World Health Organization (WHO) Framework Convention on Tobacco Control (FCTC) is the world's first public health treaty on tobacco control [1]. It provides a global response to the pandemic of tobacco-induced death and disease. The WHO FCTC urges countries to develop action plans for public policies, such as bans on direct and indirect tobacco advertising, tobacco tax and price increases, promoting smoke-free public places and workplaces, and placing health messages on tobacco packaging. It also calls for countries to establish programs for national, regional, and global surveillance. Thailand ratified the WHO FCTC on November 8, 2004.

WHO, the US Centers for Disease Control and Prevention, and the Canadian Public Health Association developed the Global Tobacco Surveillance System (GTSS) to assist all 192 WHO member states in establishing continuous tobacco control surveillance and monitoring [2]. The GTSS provides a flexible system that includes common data items but allows countries to include important unique information at their discretion. It also uses a common survey methodology, similar field procedures for data collection, and similar data management and processing techniques. The GTSS includes collection of data through three surveys: the Global Youth Tobacco Survey (GYTS) for youth, and the Global School Personnel Survey and the Global Health Professional Survey for adults.

The purpose of this paper is to use data from the GYTS conducted in Thailand in 2005 to describe baseline measures that can be used to monitor articles in the WHO FCTC and provisions of specific tobacco control legislation in Thailand. In Thailand, the survey of smoking consumption was previously part of a national survey conducted by the National Statistical Office. Therefore, the age distribution and specific questions on tobacco differed between the previous survey and the GYTS. Since the GYTS will be conducted every 3 to 4 years, we hope this will provide accurate information regarding trends in smoking by Thai youths.

### The Global Youth Tobacco Survey

In 1999, 11 countries successfully conducted and completed the GYTS [3]. To date, the GYTS has been completed by over 2 million students in 140 countries [4]. The GYTS data in this report include the following five regions in the country of Thailand: Bangkok, Central-Western, NorthEastern, Northern, and Southern. The school response rate was 100% in all sites. The student response rate was 99%. About 14,000 students participated in this survey.

The following data are presented in this report: lifetime cigarette use; initiation of smoking before age 10; likely initiation of smoking during the next year among students who had never smoked cigarettes ('never smokers'; that is, susceptibility, which is defined as the absence of a firm decision not to smoke and precedes the early experimentation stage of smoking onset; smoking onset is generally agreed to be a time-dependent, four-level process that includes preparation, early experimentation, more advanced regular but non-daily smoking, and a stable level of addiction [5]); current cigarette smoking; current use of tobacco products other than cigarettes; exposure to secondhand smoke at home; exposure to secondhand smoke in public places; desire for a ban on smoking in public places; percentage of students who were taught in school that smoking is dangerous, the reasons why young people smoke, and about the effects of smoking, such as yellowed teeth, wrinkled skin, and odor; students who have an object with a cigarette brand logo on it; percentage of smokers who wanted to stop smoking now, have tried to stop, and have received help to stop smoking; and access to and availability of cigarettes.

## Methods

The GYTS is a school-based survey, using a two-stage cluster sample design that produces representative samples of students in grades associated with ages 13 to 15 years. At the first stage, the probability of schools being selected is proportional to the number of students enrolled in the specified grades. At the second sampling stage, classes within the selected schools are randomly selected. All students in selected classes attending school the day the survey is administered are eligible to participate. Student participation is voluntary and anonymous using self-administered data-collection procedures. The GYTS sample design produces representative, independent, cross-sectional estimates for each site. For cross-site comparisons, data in this paper are limited to students aged 13 to 15 years old.

A weighting factor is applied to each student record to adjust for non-response (by school, class, and student) and variation in the probability of selection at the school and class levels. A final adjustment sums the weights by grade and sex to the population of school children in the selected grades in each sample site. SUDAAN [5], a software package for statistical analysis of correlated data, was used to compute standard errors of the estimates and produced 95% confidence intervals, which are shown as lower and upper bounds. Differences in proportions were considered statistically significant at *p *< 0.05, assessed by non-overlapping confidence intervals.

The GYTS enquired about several important tobacco-use indicators, including: current cigarette smoking (based on a response of "1 or more days" to the question, "During the past 30 days (1 month), on how many days did you smoke cigarettes?"); current use of tobacco products other than cigarettes; 'susceptibility' (that is, absence of a firm decision not to smoke) or likely initiation of cigarette smoking in the next year among never smokers (based on a negative response to the question, "Have you ever tried or experimented with cigarette smoking, even one or two puffs?" as well as a response of anything but "definitely no" to the questions, "If one of your best friends offered you a cigarette, would you smoke it?" and "Do you think you will try smoking a cigarette in the next year?") [6]; exposure to cigarette smoke in public places (based on a response of "1 or more days" to the question, "During the past 7 days, on how many days have people smoked in your presence, in places other than your home?"); one or more parents smoke cigarettes (based on a response of "both", "father only", or "mother only" to the question, "Do your parents smoke?"); one or more best friends smoke cigarettes (based on a response of "most" or "all" to the question, "Do most or all of your best friends smoke?"); in favor of banning cigarette smoking in public places (based on a positive response to the question, "Are you in favor of banning smoking in public places (such as in restaurants, in buses, streetcars, and trains, in schools, on playgrounds, in gyms and sports arenas, in discos?"); and exposure to pro-tobacco advertising and promotion, either direct or indirect (based on: a response of "a lot" or "a few" to the questions, "During the past 30 days (1 month), how many anti-smoking media messages (for example, television, radio, billboards, posters, newspapers, magazines, movies, drama) have you seen or heard?", "During the past 30 days (1 month), how many advertisements for cigarettes have you seen on billboards?", "During the past 30 days (1 month), how many advertisements for cigarettes have you seen at point of sale?", "During the past 30 days (1 month), how many advertisements or promotions for cigarettes have you seen in newspapers or magazines?"; a positive response to the questions, "Do you have something (t-shirt, pen backpack, etc) with a cigarette brand logo on it?" or "Has a cigarette company representative ever offered you a free cigarette?").

*t*-Tests were used to determine differences between subpopulations [7]. Differences between prevalence estimates were considered statistically significant if the *t*-test *p*-value was < 0.05. Differences between prevalence estimates were considered statistically significant if the *t*-test *p*-value is associated with gender and that gender most often acts as an effect modifier for smoking and related risk factors. All analyses conducted in this study were gender stratified [8].

The findings in this report are subject to at least three limitations. First, because the sample surveyed was limited to youths attending school, they may not be representative of all 13 to 15 year olds in Thailand. Second, these data apply only to youths who were in school the day the survey was administered and completed the survey. Student response was quite high in Thailand (99%; with a sample size of around 14,000), suggesting bias due to absence or non-response is small. Third, data are based on self-reports of students, who may under- or over-report their use of tobacco. The extent of this bias can not be determined in the Thailand data; however, responses to tobacco questions on surveys similar to the GYTS have shown good test-retest reliability [9].

## Results

### Prevalence

At the time of the GYTS, over 2 in 10 (23.8%) students had ever smoked cigarettes (Table 1). Boys (36.4%) were significantly more likely than girls (12.5%) to have ever smoked cigarettes. Of ever-smokers, 18.4% initiated smoking before age 10 years. Early initiation of smoking did not differ by sex. Overall, 10.1% of students in Thailand currently smoked cigarettes. Boys (17.0%) were 4 times more likely than girls (3.9%) to currently smoke cigarettes. Overall, 7.1% of students currently used tobacco products other than cigarettes. Boys (9.6%) were significantly as likely as girls (4.7%) to use other tobacco products. Among never smokers, 6.7% indicated that they were susceptible to initiating smoking during the next year. Boys were significantly more likely than girls to be susceptible to initiating smoking.

**Table 1:** Prevalence of smoking and other smoking-related behaviors, susceptibility to initiate smoking among students who had never smoked, and motivation to quit smoking among current smokers, Thailand GYTS

	**Prevalence % (95% CI)**		
	
**Students who:**	**Total**	**Male**	**Female**
Ever smoked cigarettes	23.8 (21.9–26.0) (n = 14,639)	36.4 (33.9–39.0) (n = 7,198)	12.5 (10.7–14.5) (n = 7,274)
Smoked first cigarette before age 10 years (among smokers)	18.4 (16.6–20.3) (n = 3,086)	17.4 (15.3–19.7) (n = 2,343)	19.8 (16.5–23.5) (n = 683)
Were current cigarette smokers	10.1 (9.0–11.4) (n = 14,327)	17.0 (15.1–19.1) (n = 6,948)	3.9 (3.3–4.7) (n = 7,218)
Were current users of tobacco products other than cigarettes	7.1 (6.4–7.9) (n = 14,706)	9.6 (8.5–10.7) (n = 7,254)	4.7 (4.1–5.3) (n = 7,288)
Were susceptible to initiate smoking in the next year (among never smokers)	6.7 (6.1–7.3) (n = 11,034)	9.0 (8.0–10.2) (n = 4,568)	5.2 (4.4–6.1) (n = 6,378)
Were current smokers who wanted to quit	74.1 (70.0–77.8) (n = 898)	74.3 (70.0–78.2) (n = 729)	74.1 (65.1–81.5) (n = 143)
Were current smokers who tried to quit in the year prior to the survey	82.0 (78.5–85.0) (n = 888)	83.6 (80.0–86.7) (n = 728)	78.1 (69.2–85.0) (n = 135)
Were current smokers who received help to quit in the year prior to the survey	87.6 (85.0–89.9) (n = 1,322)	87.0 (83.9–89.5) (n = 1,025)	88.2 (81.9–92.5) (n = 247)

CI, confidence interval.

### Cessation

Among students who currently smoked cigarettes, 74.1% reported that they want to stop smoking now; 82.0% stated that they had tried to stop smoking during the past year but had failed; and 87.6% reported that they had received help to stop smoking (Table 1). There were no differences by sex for any of these indicators.

### Exposure to secondhand smoke

Almost half (47.9%) of students in Thailand reported that they were exposed to smoke from others in their home; 68.5% reported that they were exposed to smoke from others in public places; and 91.0% thought smoking should be banned in public places (Table 2). Girls were significantly more likely than boys to support the ban on smoking in public places.

**Table 2:** Prevalence of factors that influence smoking and other smoking-related behaviors, Thailand GYTS, 2005

	**Prevalence % (95% CI)**		
	
**Students who:**	**Total**	**Male**	**Female**
Were exposed to smoke from others at home	47.9 (46.6–49.3) (n = 14,804)	47.4 (45.6–49.2) (n = 7,306)	48.0 (46.0–50.0) (n = 7,319)
Were exposed to smoke in public places	68.5 (67.2–69.9) (n = 14,805)	68.0 (66.2–69.7) (n = 7,308)	68.7 (67.1–70.3) (n = 7,322)
Thought smoking should be banned in public places	91.0 (90.1–91.8) (n = 14,719)	88.0 (86.7–89.2) (n = 7,252)	93.7 (92.6–94.6) (n = 7,307)
Had an object with a tobacco brand logo on it	39.3 (37.9–40.6) (n = 14,390)	41.7 (40.1–43.3) (n = 7,110)	36.9 (35.0–38.8) (n = 7,146)
Were ever offered free cigarettes by a tobacco company representative	9.0 (8.2–9.8) (n = 14,396)	11.4 (10.1–12.7) (n = 7,098)	6.6 (5.7–7.6) (n = 7,158)
Usually bought cigarettes in a store	36.8 (33.6–40.1) (n = 1,463)	36.9 (33.1–40.8) (n = 1,119)	35.9 (29.9–42.5) (n = 293)
Were not refused purchase because of their age in the month prior to the survey (among students who usually bought cigarettes in a store)	30.8 (25.7–36.5) (n = 422)	29.5 (24.1–35.6) (n = 342)	39.1 (27.2–52.4) (n = 69)
Were taught the dangers of smoking in the year prior to the survey	65.2 (63.9–66.4) (n = 14,599)	62.2 (60.7–63.7) (n = 7,206)	68.1 (66.3–69.9) (n = 7,241)
Discussed why people their age use tobacco in the year prior to the survey	29.5 (28.0–31.1) (n = 14,757)	28.3 (26.6–30.0) (n = 7,293)	30.7 (28.4–33.1) (n = 7,306)
Were taught about the effects of smoking tobacco such as yellowed teeth, wrinkled skin, and odor	60.7 (59.3–62.0) (n = 14,751)	56.5 (54.7–58.2) (n = 7,292)	64.7 (62.9–66.5) (n = 7,306)

CI, confidence interval.

### Exposure to indirect advertising

Almost 4 in 10 (39.3%) students in Thailand reported that they had an object (that is, hat, t-shirt, knapsack, and so on) with a cigarette or tobacco brand logo on it (Table 2). Boys were significantly more likely than girls to have an object with a logo on it. Students were asked if they had been offered free cigarettes by a tobacco company representative at any time. Overall, 9.0% of students had been offered free cigarettes. Boys were significantly more likely than girls to have been offered free cigarettes.

### Access and availability

Almost four in 10 (36.8%) students who currently smoked reported that they 'usually' bought their cigarettes in a store (Table 2). Current smokers who usually buy their cigarettes in a store were asked if they had been refused purchase because of their age in the month prior to the survey. Approximately 3 in 10 (30.8%) reported they had not been refused purchase because of their age. Purchasing in a store and not being refused purchase because of their age did not differ by sex.

### Taught in school about the dangers of tobacco

Students were asked if, during the past school year in classes, they had been taught about the dangers of tobacco, discussed the reasons why young people smoke, and been taught about the specific health effects of tobacco (Table 2).

More than 6 in 10 (65.2%) students in Thailand reported that they had been taught about the dangers of tobacco; 29.5% reported that they had discussed reasons why young people use tobacco; and 60.7% were taught about the specific health effects of tobacco use. Girls were significantly more likely than boys to have been taught about the dangers of tobacco use and about the effects of smoking.

## Discussion

As a country with advanced tobacco control policies and initiatives, tobacco control advocates in Thailand expect that Thai youths will have a lower rate of tobacco consumption compared to other countries.

For the past 10 years, Action on Smoking or Health (ASH-Thailand) has implemented a vigorous campaign among Thai youths to inform them about the harmful effects of smoking. These campaigns have included using actors/actresses, sportsmen/sportswomen, and Miss Thailand as role models to talk to students in primary, secondary and high schools around the country. Results from the GYTS suggest tobacco control efforts in Thailand may need to be increased and expanded. One in 10 students in Thailand (10.1%) currently smoked cigarettes, which is higher than the overall GYTS rate (8.9%) and the average rate (5.8%) in the South-East Asia Region (SEARO) of WHO [10]. This is surprising as Thailand has been a leader in tobacco control both internationally and in SEARO. Furthermore, the differential between boys and girls in the susceptibility of never smokers to initiate smoking in the next year (that is, boys are 1.7 times more susceptible than girls) is significantly less than the differential between rates of current cigarette smoking (that is, the rate of smoking for boys is 4.3 times higher than that for girls). This may be an indication that cigarette smoking will soon be increasing among young girls.

In 1992, Thailand passed the Non-smokers' Health Protection Act, which banned smoking in public places [11]. Since 1992, the Ministry of Public Health has periodically added new public places to be designated as tobacco-free. Unfortunately, while the law is strong, enforcement of the provisions is weak and the penalty is very light; therefore, adherence to the law is low. The Ministry of Public Health needs to develop and implement effective enforcement strategies.

The Thailand Tobacco Products Control Act of 1992 includes a comprehensive ban on advertising. According to this act, there should be no advertisement of tobacco products in any form. But the trans-national tobacco industry tries indirect advertising by giving away free objects with tobacco brand logos on them, such as t-shirts, backpacks, hats, and so on, which is illegal. Results of the GYTS indicated that almost 4 in 10 students had an object (t-shirt, hat, knapsack, sticker, and so on) with a tobacco company logo on it. Young people who have an object with a tobacco company logo on it are 'mobile advertising billboards' for the tobacco industry.

The 1992 law also prohibits selling tobacco products to youths under 18 years of age [11]. GYTS data show that more than 3 in 10 current smokers usually buy their cigarettes in a store and about 3 in 10 were not refused purchase because of their age during the month prior to the survey. About 1 in 10 was offered free cigarettes by a tobacco company representative. Clearly, enforcement of this law is a major issue facing Thailand as well as implementing the prohibition of stores selling cigarettes within 500 meters of schools.

Seven in 10 current smokers wanted to stop smoking and over 8 in 10 have tried to stop during the past year but failed. These findings suggest a need to develop, pilot test, evaluate, and implement effective smoking cessation programs for youths. Once effective programs have been identified, they need to be made widely available throughout Thailand.

Overall, more than 6 out of 10 students in Thailand reported that, during the past school year, they had been taught about the dangers of smoking and about one-third had discussed reasons why people their age smoke. These rates are high but the Ministry of Public Health and Ministry of Education should work together to evaluate the effectiveness of these programs and develop and implement new programs as needed.

## Conclusion

The data from the GYTS provide valuable information regarding the effectiveness of tobacco control policies in Thailand. Thailand passed two important laws regarding tobacco control in 1992. These two laws, the Tobacco Products Control Act of 1992 and the Non-smokers' Health Protection Act of 1992, are milestones for tobacco control advocates in Thailand. These laws include strong language for tobacco control, but enforcement has been weak and circumventions and violations are still very common [12]. The Ministry of Public Health needs to develop strategies on how to further implement and effectively enforce the laws.

Thailand needs to use the GYTS data to assist in developing a national tobacco control policy and plan of action as recommended in the WHO SEARO strategy document [13] and the WHO FCTC. Previous studies have shown that demand reduction measures, primarily those that increase the price of tobacco, are effective in reducing consumption among adults who smoke and can encourage and support cessation among adults [14,15].

Since young people are price-sensitive [16], price increases have the added benefit of significantly reducing initiation of tobacco use and consumption among young people. Tobacco companies have challenged the effectiveness of raising taxes by arguing that increased taxes will lead to increased smuggling, which will result in less revenue for governments. However, according to the World Bank, the experience of a large number of high-income countries shows that, even in areas that experience a high level of smuggling, tax increases bring increased revenues and reduce cigarette consumption [17]. Cigarette smuggling is addressed in Article 15 of the WHO FCTC, which calls for countries to reduce smuggling through national, regional, and global action [1].

In addition to demand-reduction measures, comprehensive tobacco control programs often include non-price interventions, such as: restrictions on smoking in public places and work places; a complete ban on advertising and promotion by tobacco companies; dissemination of information on the health consequences of smoking through various media, such as prominent warning labels on cigarette packets and counter-marketing campaigns; and development and implementation of school-based educational programs in combination with community-based activities [14,18].

Development of an effective comprehensive tobacco control program will require careful monitoring and evaluation of existing programs and likely development of new efforts. Components of a comprehensive tobacco control program could include higher taxes, comprehensive bans on the promotion and advertising of tobacco, prominent warning labels, de-regulated access to nicotine-replacement therapies, and tight controls on smuggling [14]. Results from the GYTS have suggested that the effectiveness of tobacco control policies in Thailand, as well as in other countries around the world, will have to be a collaboration, and involve coordination and cooperation between various government agencies, and partnerships between government and non-government organizations. Additionally, collaboration, coordination and cooperation between countries are necessary to reduce problems of cross-border advertising and cigarette smuggling. The synergy between Thailand's leadership in passing the 1992 laws, in ratifying the WHO FCTC, and in supporting the conduct of the GYTS offers Thailand a unique opportunity to develop, implement and evaluate comprehensive tobacco control policy that can be most helpful to the country.

## List of abbreviations used

FCTC: Framework Convention on Tobacco Control; GTSS: Global Tobacco Surveillance System; GYTS: Global Youth Tobacco Survey; SEARO: South-East Asia Region; WHO: World Health Organization.

## Competing interests

The authors declare that they have no competing interests.

## Authors' contributions

NS contributed to data collection, drafting, writing and revising the manuscript, and getting final approval of the version to be published. CT contributed to data collection and planning. KR contributed to proof-reading and editing of the manuscript. CWW contributed to conception and design of the study, analysis and interpretation of data, and revising of the manuscript. NRJ contributed to analysis and interpretation of data. SA contributed to editing and revising the manuscript. JL contributed to analysis and interpretation of data.
